# Positive Response to Fluorouracil and Oxaliplatin in Signet Ring Cell Adenocarcinoma of the Bladder Presenting With Retroperitoneal Fibrosis

**DOI:** 10.7759/cureus.9322

**Published:** 2020-07-21

**Authors:** Sanjay Hinduja, Haifaa Abdulhaq, Salimah Valliani, Mir M Ali

**Affiliations:** 1 Hematology/Oncology, University of California San Francisco, Fresno, USA

**Keywords:** signet cell bladder cancer, bladder cancer

## Abstract

Bladder adenocarcinoma is an uncommon type of bladder cancer. Signet ring cell pathology is a rare subtype of bladder adenocarcinoma. Global incidence rates of signet ring cell adenocarcinoma of the bladder have not been established. Management of signet cell bladder cancer is challenging as it is aggressive in behavior with frequent relapse despite chemotherapy. Here we present a case of stage IV signet cell bladder cancer with retroperitoneal fibrosis treated with FOLFOX (folinic acid, 5-fluorouracil, oxaliplatin) regimen with a complete durable response.

## Introduction

Primary signet cell bladder cancer is a rare tumor type. It accounts for up to 0.5-2% of all primary bladder cancers [1]. A literature review yields fewer than 100 published case reports. There are no established guidelines for chemotherapy in these patients, and treatment is based on case reports [2]. The prognosis of primary signet cell bladder cancer is generally poor due to the advanced stage of disease at diagnosis. This cancer needs to be differentiated from metastatic cancer from other primary sites, making diagnosis challenging. Here we report a case of a patient who presented to our practice with retroperitoneal fibrosis and was diagnosed with metastatic signet cell adenocarcinoma of the bladder. We treated the patient with FOLFOX (folinic acid, 5-fluorouracil [5FU], oxaliplatin) regimen and had a clinical and radiological response.

## Case presentation

**Figure 1:**
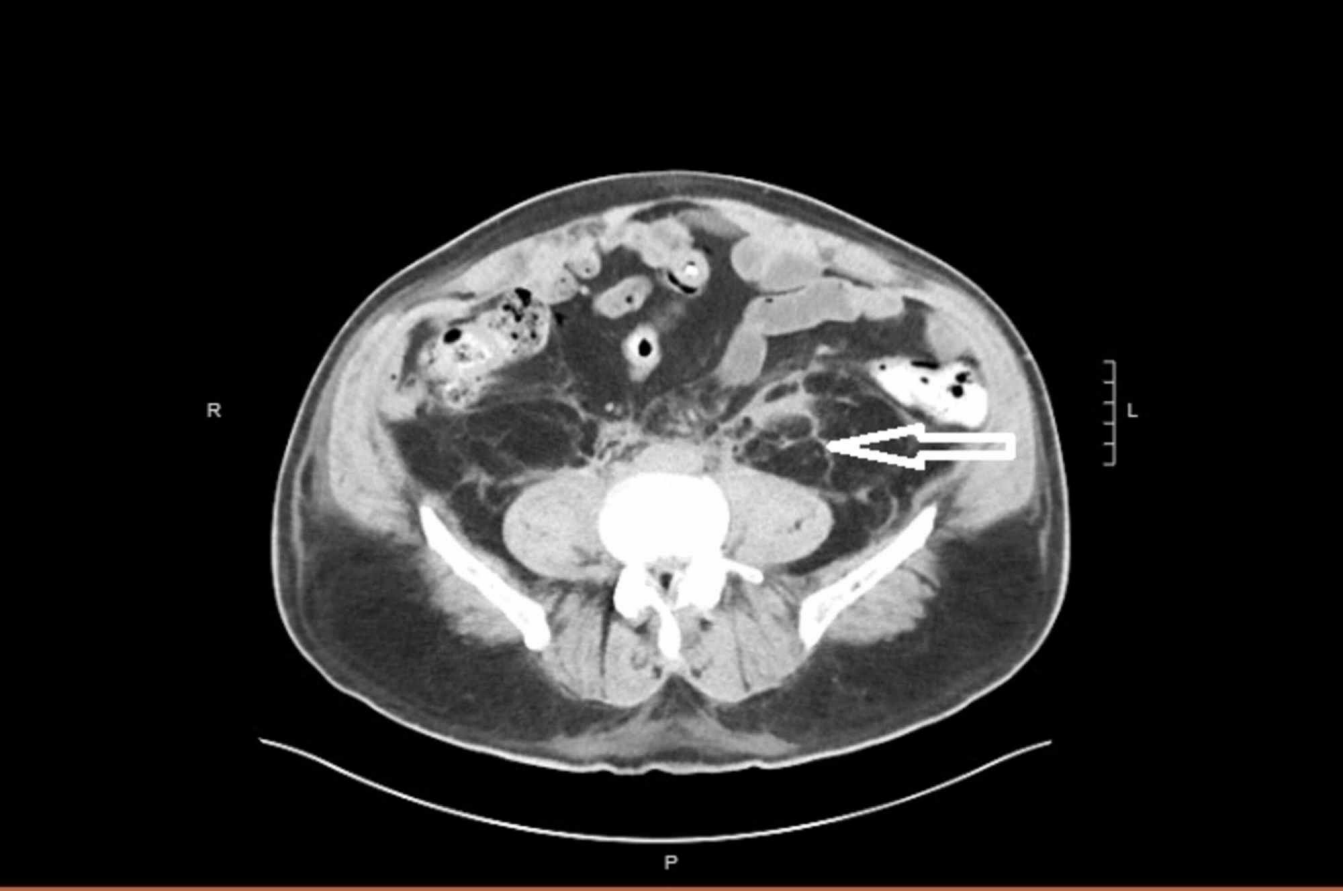
Cross-sectional CT shows the presence of retroperitoneal fibrosis with the depicted arrow.

**Figure 2:**
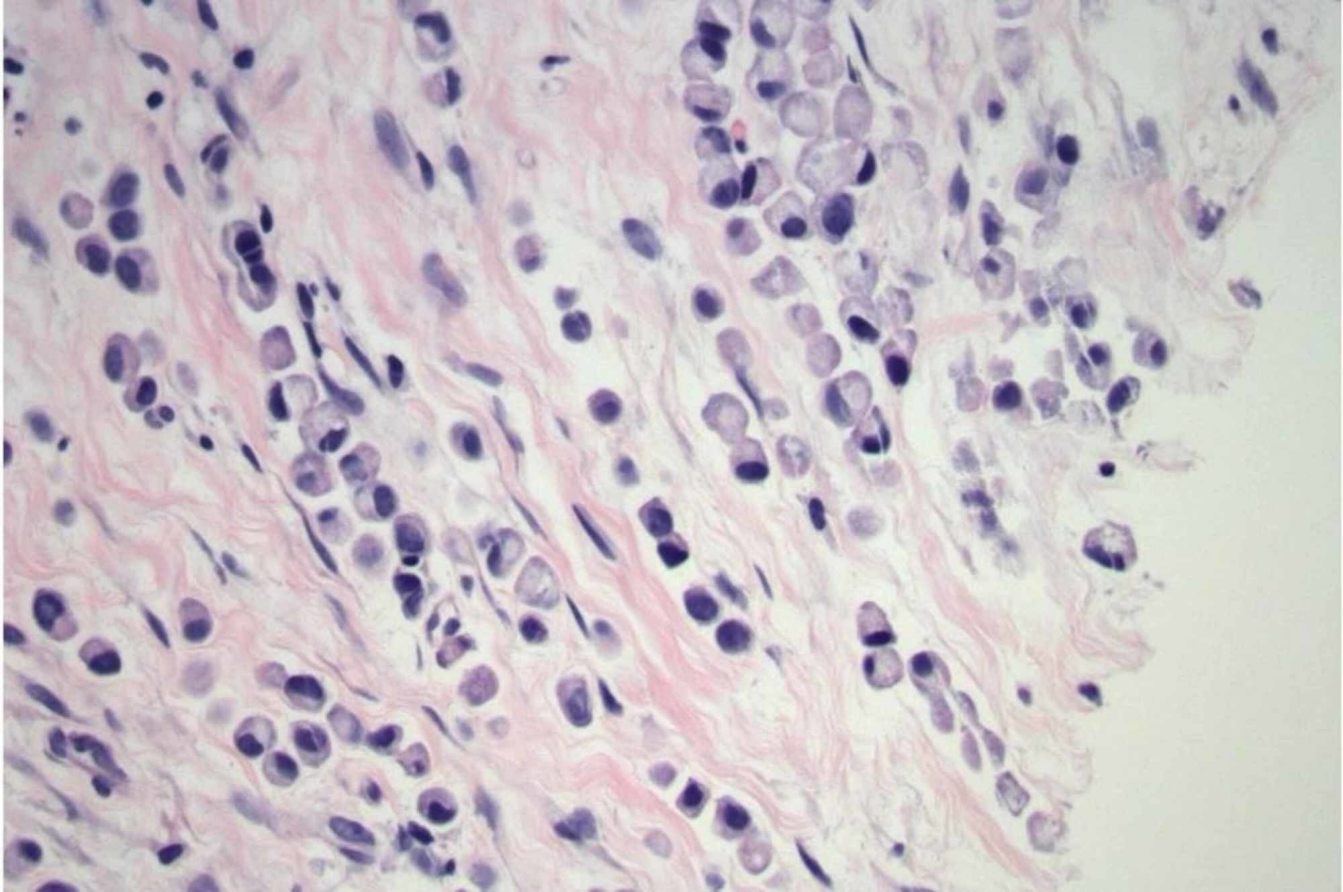
Signet cell pathology demonstrated on hematoxylin and eosin stain.

We present the case of a 66-year-old male with a past medical history of non-muscle invasive bladder cancer (stage I) diagnosed one year ago at an outside facility. He was treated with transurethral resection of the bladder tumor. He presented to our institution with worsening abdominal pain, low appetite, and new-onset fatigue for two months. CT imaging of the abdomen showed the presence of retroperitoneal fibrosis along with right-sided hydronephrosis (Figure 1). There was no spread of cancer identified in the chest. The patient underwent a right nephrostomy tube placement. A retroperitoneal biopsy was obtained, which showed the presence of poorly differentiated neoplasm with signet ring cell features (Figure 2). The patient subsequently underwent esophagogastroduodenoscopy (EGD), which was unrevealing for a primary gastric tumor. Colonoscopy was attempted; however, it was unsuccessful as the colonoscope could not be advanced due to severe pelvic fibrosis. At this point in time, we did not have the patient’s outside records, leaving us with a diagnostic conundrum. A “cancerTYPE ID” test was ordered. This test uses real-time PCR to evaluate the expression of 92 genes in the patient’s tumor and compares these gene expression patterns to a proprietary database to help identify the site of the primary tumor. In this case, the test pointed toward primary bladder cancer. Eventually, the patient’s previous biopsy results were located from the outside facility and compared with the current biopsy. They were found to be identical. Pathology showed the presence of signet cells invading into the retroperitoneum. Immunohistochemistry results showed the presence of cytokeratin 7 and cytokeratin 20. Caudal-type homeobox transcription factor 2 protein (CDX2) was negative. This pattern is suggestive of signet ring cell pathology. We concluded that the patient now had metastatic adenocarcinoma of the bladder. Serum markers were obtained, which showed elevated carcinoembryonic antigen (CEA) at 80.5 ng/mL and CA 19-9 at 34,890 U/mL. At the commencement of systemic chemotherapy, the patient’s functional status was Eastern Cooperative Oncology Group (ECOG) 4. The patient was offered systemic therapy after a discussion of risks and benefits given his poor functional status. He elected to undergo systemic chemotherapy.

**Figure 3:**
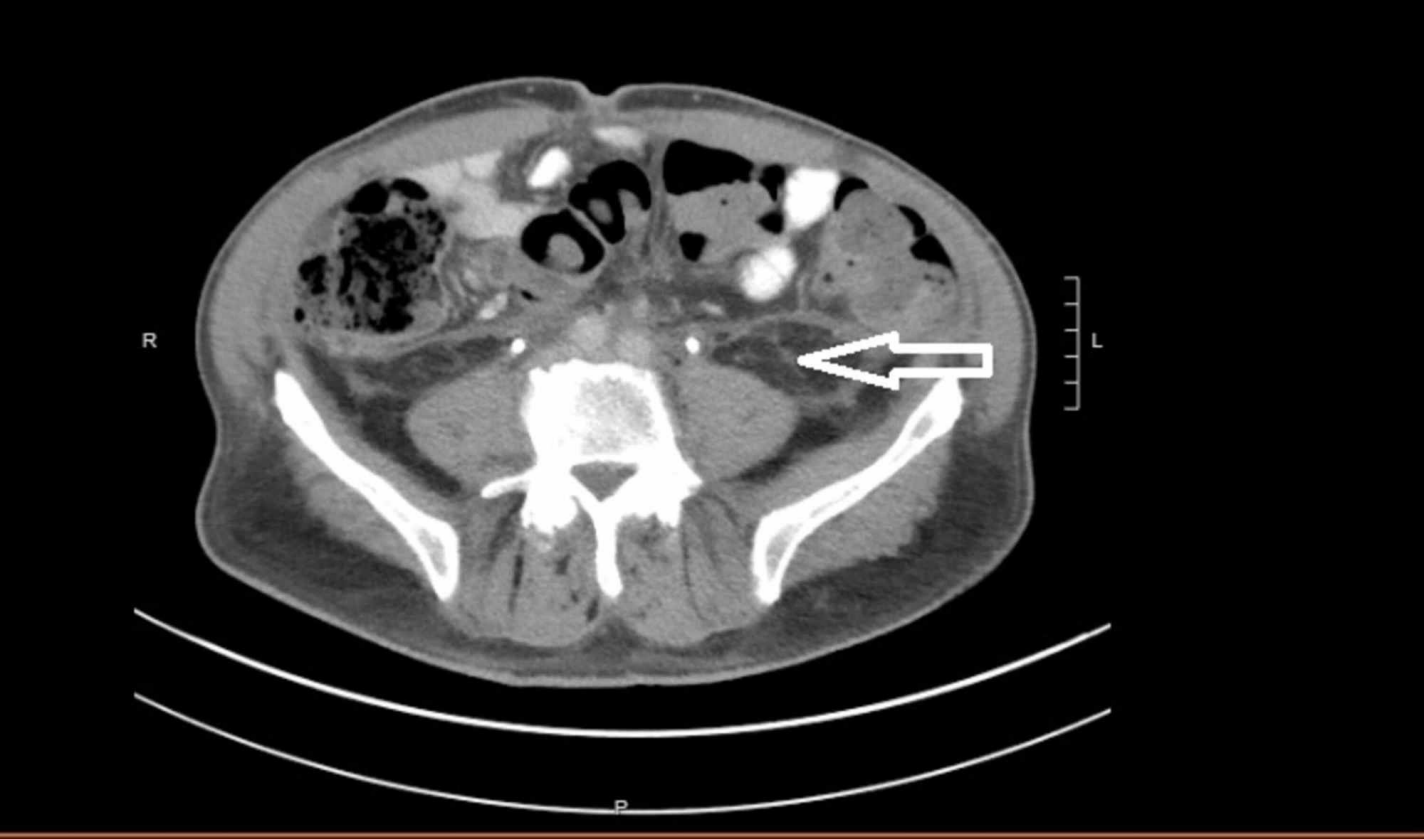
CT shows near resolution of retroperitoneal fibrosis depicted with the arrow.

The patient was started on systemic therapy with FOLFOX (5FU 400 mg/m^2^ IV bolus on day 1, 5FU IV with a total dose of 2,400 mg/m^2^ continuous infusion over 46 hours on days 1 to 2, folinic acid 400 mg/m^2^ IV on day 1, oxaliplatin 85 mg/m^2^ IV on day 1). He had a good clinical response with improvement in ECOG score to 1 after 12 cycles. The patient experienced moderate side effects such as nausea and vomiting along with grade 2 neuropathy related to oxaliplatin toxicity, which required lowering the dose of oxaliplatin to 70 mg/m^2^ after eight cycles. After nine cycles of FOLFOX, the patient’s CEA normalized to 3.3 and CA 19-9 reduced to 154. CT of the abdomen was obtained after 12 cycles of FOLFOX, which showed near resolution of retroperitoneal fibrosis (Figure 3). The patient was then placed on 5FU with leucovorin and remained in remission for three months. This was followed by recurrence with malignant ascites requiring frequent paracentesis. Second-line therapy with FOLFIRINOX (5FU, leucovorin, oxaliplatin, irinotecan) was considered. The patient had been on chemotherapy for 10 months. Despite having initial clinical improvement, he had a disease relapse, which affected his quality of life greatly. Therefore, he chose to enroll in hospice.

## Discussion

This case presented a particular challenge with the patient receiving part of his care outside our health system. The pathology showed the presence of signet ring cell appearance, which required a thorough evaluation of the gastrointestinal tract for malignancy. However, no gastrointestinal primary neoplasia was found on EGD. Serum markers were obtained to monitor response to therapy. The literature does not support the use of tumor markers in bladder cancer; however, Pall et al. showed that CA 19-9 can be elevated in urothelial carcinoma [3]. The near normalization of CA 19-9 suggests the possible utility of this tumor marker in bladder cancer.

Treatment of signet cell bladder cancer has no established guidelines [4]. This cancer is aggressive in nature and tends to be metastatic at diagnosis. Cystectomy remains a pillar of treatment for early-stage signet cell adenocarcinoma of the bladder [5]. Retroperitoneal fibrosis has been described as a rare presentation of signet ring cell adenocarcinoma of the bladder by Iqbal et al. [6]. Our case is unique as it would be the first to describe the chemotherapeutic treatment of retroperitoneal fibrosis as a result of the signet ring cell adenocarcinoma of the bladder. A case series published by Dayyani et al. shows the use of various chemotherapy regimens that have been tried such as gemcitabine with cisplatin and DDMVAC (dose-dense methotrexate, vinblastine, doxorubicin, and cisplatin) [7]. All of these have been met with limited success as this cancer is generally resistant to chemotherapy regimens used for urothelial carcinoma of the bladder [4]. We chose to treat the patient with FOLFOX regimen as the signet cell pathology of bladder is analogous to gastric cancer. A case report by Akamatsu et al. showed that 5FU-based regimen does have activity in signet cell bladder cancer much like signet cell gastric cancer [8]. Hamakawa et al. showed the efficacy of S-1 (tegafur/gimeracil/oteracil) in combination with cisplatin in the adjuvant setting. This further supports the utility of combining 5FU-based regimens in combination with platinum agents in signet cell adenocarcinoma of the bladder.

## Conclusions

This case highlights the unique clinical challenges in diagnosing and treating signet cell bladder cancer. There is a paucity of data in the treatment approach of signet cell bladder cancer. Our case supports the use of FOLFOX chemotherapy in treating signet cell bladder cancer. Further randomized controlled trials are needed to formulate an optimal approach for the treatment of this disease.
